# Inoculation for Rabies and Hydrophobia. A Study of the Literature of the Subject

**Published:** 1887-09

**Authors:** 


*Inoculation for Rabies and Hydrophobia. A Study of the Literature of the Subject.* By Surgeon-General C. A. GORDON, M.D., C.B. Pp. 127. London: BAILLIÈRE, TINDALL, and COX. 1887.

We are in doubt whether this is seriously intended as a scientific work. The author describes it on the title-page as a study of the Literature of Inoculation for Rabies and Hydrophobia. Now evidence may be collected from the student in the workshop, or from gossip in the market-place. Our author has chosen the latter source. The literature studied and quoted is that ordinarily found on the table of the reading-room of a London Club. We have quotations from *The Times* and *The Field*—these have some weight; and we have extracts from *The Standard*, *The Morning Post*, *The Telegraph*, *The Chronicle*, *The Daily News*, *The Spectator*, and *The Evening Standard*—these we can tolerate; but what shall we say of the value of scientific evidence culled from *The Globe*, *The World*, *Vanity Fair*, *The Referee*, *The Zoophilist*, and (save the mark!) *The Pall Mall Gazette?*

With the exception of *The Medical Press and Circular* and *The British Medical Journal*, modern scientific journals have been almost ignored. We must therefore decline to regard this work as a "study of the Literature of the subject of Inoculation for Rabies and Hydrophobia," and, as scientific men, disregard conclusions derived from unscientific sources.

We fully believe that Surgeon-General Gordon is actuated by the best of motives, and that he may honestly "disclaim all partizanship in reference to the question he endeavours to elucidate, further than what is justified by the data to which he has had access" (Preface). But the data to which he has had access are not the accessible data.

